# Insanity of English People

**Published:** 1897-06

**Authors:** 


### Insanity of English People.

Professor J. Holt Schooling, Fellow of the Royal Statistical Society, has ended his investigation and concludes that one in every 360 of the population of Great Britain has some form of insanity. He gives facts to show that in every 10,000 of the English and Welsh population, 31.4 people are lunatics. In every 10,000 of the Scotch population, 33.6 people are lunatics. In every 10,000 of the Irish population 40.3 people are lunatics. As among the chief causes, with the percentage of each in classes of a hundred, he gives the following: Drink, 33.6; domestic troubles, 15.1; mental anxiety, 13.4; old age, 13.2; adverse circumstances, 13; accidents, 6.5; religious excitement, 4; love affairs, 3.2.

